# Comparing the efficacy of therapeutic Thai acupressure on plantar acupoints and laser cane therapy on freezing of gait in Parkinson’s disease: a randomized non-inferiority trial

**DOI:** 10.3389/fneur.2024.1327448

**Published:** 2024-01-29

**Authors:** Yuka Miyahara, Onanong Phokaewvarangkul, Stephen Kerr, Chanawat Anan, Haruki Toriumi, Roongroj Bhidayasiri

**Affiliations:** ^1^Doctor of Philosophy Program in Medical Sciences (International Program), Faculty of Medicine, Chulalongkorn University, Bangkok, Thailand; ^2^Chulalongkorn Centre of Excellence for Parkinson’s Disease and Related Disorders, Department of Medicine, Faculty of Medicine, Chulalongkorn University, and King Chulalongkorn Memorial Hospital, Thai Red Cross Society, Bangkok, Thailand; ^3^Wat Pho Thai Traditional Medical School, Bangkok, Thailand; ^4^Biostatistics Excellence Centre, Faculty of Medicine, Chulalongkorn University, Bangkok, Thailand; ^5^The Kirby Institute, University of New South Wales, Sydney, NSW, Australia; ^6^Department of Acupuncture, Shonan Keiiku Hospital, Fujisawa, Japan; ^7^Toriumi Acupuncture Clinic, Tokyo, Japan; ^8^The Academy of Science, The Royal Society of Thailand, Bangkok, Thailand

**Keywords:** Parkinson’s disease, neurologic gait disorders, freezing of gait, quality of life, cues, acupressure, proprioception

## Abstract

**Background:**

ON-freezing of gait (ON-FOG) in Parkinson’s disease (PD), often resistant to medication, is linked to sensory deficits and proprioceptive impairment, and results in falls and reduced life quality. While visual cues from a laser cane (LC), which rapidly accesses the motor cortex, are commonly used to compensate for proprioceptive impairment, increased visual reliance may be affected by disease progression. Emerging evidence suggests that modulation of peripheral sensory processing may alleviate ON-FOG, and therapeutic Thai acupressure (TTA) may be a solution. This study aims to evaluate the effect of TTA in alleviating ON-FOG and compare its effectiveness to LC in patients with PD.

**Methods:**

This open-label, non-inferiority trial randomized 90 PD patients with ON-FOG equally into three arms: TTA for plantar nerve stimulation for 96 s, LC for visual cueing, and sham control (SC). Stride length was the primary non-inferiority endpoint [non-inferiority margin: lower limit of 95% confidence interval (CI) above −10 cm in mean change difference in pre- and immediately post-intervention in TTA versus LC (one-sided)]. Secondary outcomes included FOG episodes, double support time, velocity, cadence, step length, timed up and go (TUG) test, and visual analog scale (VAS) score.

**Results:**

TTA showed non-inferiority to LC in stride length (mean = −0.7 cm; 95% CI: −6.55; 5.15) (one-sided). The improvements with TTA and LC versus SC were comparable between (mean = 13.11 cm; 95% CI: 7.26; 18.96) and (mean = 13.8 cm; 95% CI: 7.96; 19.65) (one-sided). Secondary outcomes favored TTA and LC over SC with improved FOG, velocity, step length, and VAS scores, while only TTA resulted in improved double support time, cadence, and TUG test results. No complications occurred.

**Conclusion:**

The efficacy of TTA, which improves stride length, is non-inferior to that of LC and consequently alleviates FOG comparable to LC. TTA might enhance proprioceptive function and reduce visual dependence. Therefore, TTA, characterized by its non-invasive, simple, and safe techniques, is a potential non-pharmacological alternative for ON-FOG treatment and might enhance overall quality of life. However, further research into the mechanism, efficacy, and utilization of TTA is essential.

**Clinical trial registration:**

https://www.thaiclinicaltrials.org/show/TCTR20200317001, identifier TCTR20200317001.

## 1 Introduction

In Parkinson’s disease (PD), freezing of gait (FOG) is a disabling motor symptom. This phenomenon is described as “a brief, episodic absence or marked reduction in forward progression of the feet despite the intention to walk,” resulting in falls and reduced quality of life (1). As PD progresses, the prevalence of FOG also increases. Although its milder forms can be recognized early, over half of patients with PD will ultimately experience FOG (2). FOG presents in two distinct forms: ON-FOG, which occurs when the medication takes full effect (ON-state), and OFF-FOG, which happens when the medication efficacy wanes. Notably, ON-FOG is particularly challenging owing to its resistance to many medications (3).

Although the underlying causes of ON-FOG remain under investigation, it is considered a dysfunction either in dopaminergic function or in extra-striatal regions, where projections of the cortex and cerebellum where motor and sensory signals interact (2, 4, 5). However, prevalent theories underscore sensory deficits in gait and balance control from peripheral side (6). A significant loss of Aβ cutaneous mechanoreceptors may link to peripheral neuronal degeneration to cause peripheral deafferentation to central nerve system on PD patients (7). A key element is impaired proprioceptive feedback (8). For instance, during the ON state, FOG increases in situations relying heavily on proprioception—such as navigating doorways in the dark (9). A combination of inadequate proprioceptive feedback and visuomotor disruptions might contribute to FOG (5, 9). Insufficient proprioceptive feedback can confuse the position sense attributed to space perception, thereby hindering motor planning and leading to shortened stride lengths and the onset of FOG (10). Consequently, stride length is often a FOG indicator (11, 12). To counter proprioceptive deficits, visual cues, notably from a laser cane (LC), have gained attention as effective gait-improvement tools (13). These cues, produced by laser light, either sharpen focus on a target or facilitate movement, aiding patients in modulating stride length to counter ON-FOG (13–15). Thailand has even adopted LC as a standard complementary and alternative medicine (CAM) treatment (14, 16). However, there are some concerns that increased reliance on visual cues, against a backdrop of PD-induced visual dysfunction, might exacerbate FOG in the long term (15, 17–19). The need for alternative treatments that address the core issue of proprioceptive impairment is underscored.

Emerging evidence supports the efficacy of peripheral sensory processing manipulations in mitigating ON-FOG (20). Tools such as metallic mechanical stimulators and silicone pads have garnered attention. Previous studies suggest their potential in not only reducing the severity of FOG but also in refining balance control and various gait metrics, such as stride length (21–23). The principle behind this is enhancing afferent input to the central pattern generators (CPGs) from the periphery, which in turn improves sensorimotor function.

Traditional Asian medical treatments may have an underlying scientific basis for their treatment of PD (24). Acupressure, a CAM sensory manipulation, holds potential in the treatment of ON-FOG. In Thailand, the deep therapeutic Thai acupressure (TTA) technique has recognized benefits in mitigating musculoskeletal disorders and pain (25–27). TTA involves applying thumb pressure on specific acupoints situated along meridian lines. The pressure varies in intensity, tailored to the pain threshold of each recipient, and is typically repeated multiple times for each point (26, 28, 29). Such pressure potentially stimulates proprioceptors including spindle cells and Golgi tendon organs, harmonizing muscle tension and neuromuscular excitability and boosting proprioceptive feedback (26, 28, 29). TTA provides benefits that include improved balance, foot sensation in diabetic neuropathy patients, and even increased muscle strength in ON-state patients with PD (28, 30). These improvements are primarily attributed to enhanced proprioception (28, 30). However, the efficacy of TTA for managing ON-FOG is still unclear. Similarly, although plantar stimulations appear promising, their potential in alleviating increased visual dependency is yet to be determined. Here, it has to be noted that patients with PD rely heavily on visual cues, because they provide the most powerful and rapid access to visuomotor function to compensate for the inadequate proprioceptive feedback and facilitate the initial movement (31). Therefore, we hypothesized that TTA might alleviate ON-FOG with a non-inferior magnitude compared with LC in terms of the immediate effect.

Taken together, we aimed to compare the efficacy of TTA with that of LC in treating ON-FOG in patients with PD, particularly regarding the immediate effect. Toward this goal, we adopted a non-inferiority trial design that involved three arms: TTA, LC, and a placebo-controlled sham arm.

## 2 Materials and methods

### 2.1 Study design and participants

This study was an interventional, randomized, open-label, three-armed parallel-group, controlled, non-inferiority trial. The trial enrolled patients diagnosed with PD using the United Kingdom Parkinson’s Disease Society Brain Bank criteria at the Chulalongkorn Centre of Excellence for Parkinson’s Disease and Related Disorders at King Chulalongkorn Memorial Hospital in Bangkok, Thailand (ChulaPD). Only patients whose condition was both clinically verified by movement disorder specialists and had medically intractable ON-FOG were eligible. The inclusion criteria were age ≥40 years, ability to walk a minimum of 10 m independently, and showing ON-FOG symptoms even after maintaining a consistent medication regimen for at least 3 months. The exclusion criteria were as follows: inability to walk unaided and exhibiting any of the following: utilization of deep brain stimulation, presence of other neurological disorders apart from PD, dementia as identified by a score exceeding 1 on the Unified Parkinson’s Disease Rating Scale (UPDRS)-Part I item 1, acute visual impairments, severe depression, diabetic-induced peripheral neuropathy, active foot skin conditions, systolic blood pressure above 140 mmHg, and diastolic pressure above 90 mmHg.

FOG was determined based on a minimum score of 2 on item 14 related to walking freeze in the UPDRS-Part II (32). To evaluate the medical intractability of FOG, patients were requested to execute 540° turns both left and right at their regular and highest speeds during their ON-state and while on their typical medication.

This study was approved by the Human Ethics Committee of the Faculty of Medicine, Chulalongkorn University (Institutional Review Board No. 211/62) and was conducted according to the tenets of the Declaration of Helsinki. The study is also registered with the Thai Clinical Trial Registry (TCTR20200317001). All participants provided informed consent before participation.

### 2.2 Experimental protocol

**Figure 1:**
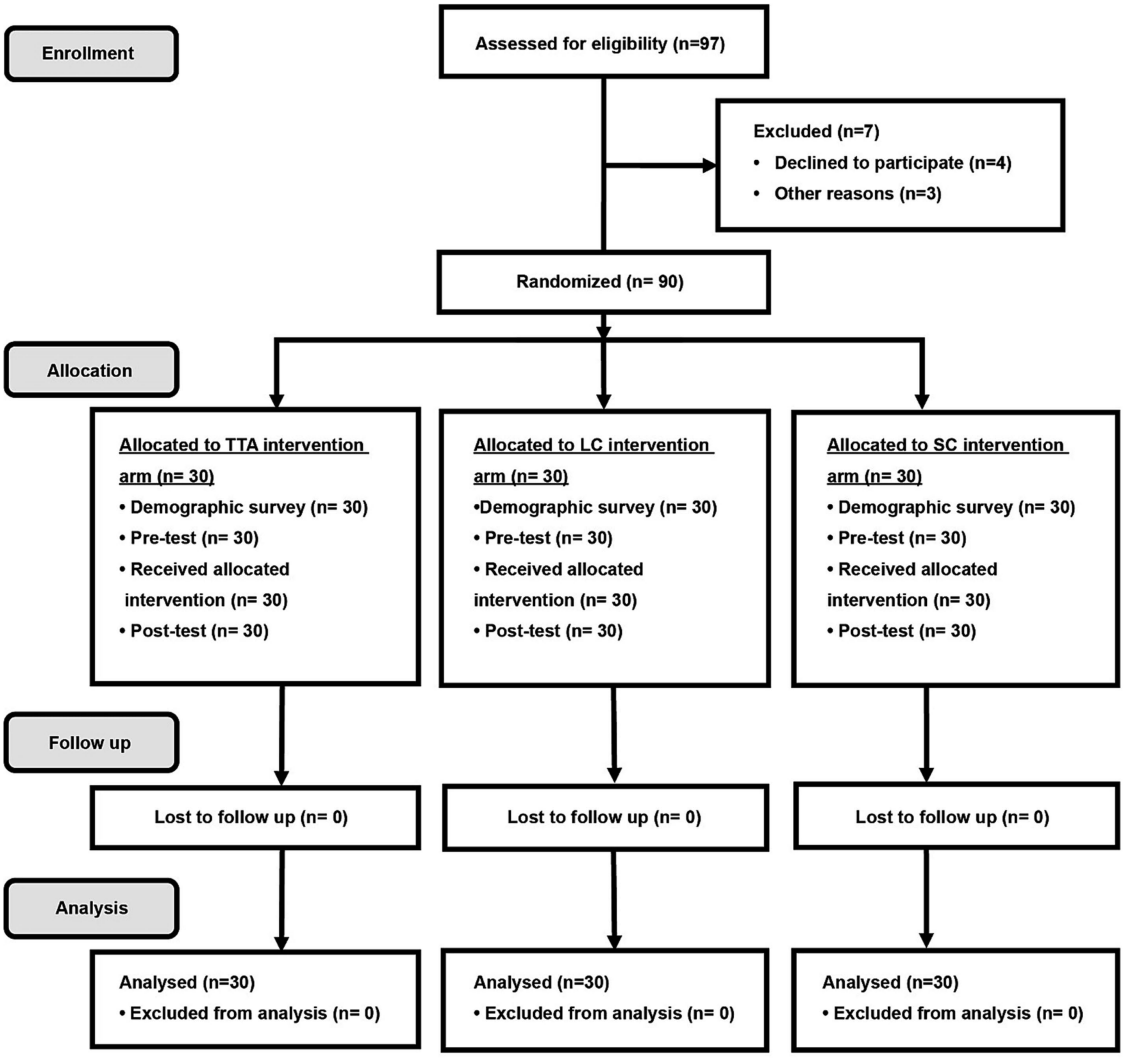
CONSORT randomization flow diagram. TTA, therapeutic Thai acupressure; LC, laser cane; SC, sham-control of light touch.

The participants were randomized in a 1:1:1 ratio into three arms: stimulation of plantar nerves via TTA as a novel active treatment, visual cues through LC walking as a standard active treatment, or light touch on the plantar surface as a placebo internal sham control (SC) (33). The allocation was conducted using sex-stratified permuted block randomization (Figure 1). Evaluations took place in the ON-state at 30–60 min after taking their usual dopaminergic medication. To establish baseline characteristics, movement disorder specialists assessed the Hoehn & Yahr stage, UPDRS-Part III, levodopa equivalence dosage, and disease duration (32, 34). The freezing of gait questionnaire (FOG-Q) was administered to determine daily life FOG episodes (35). The FOG-Q has 6 items with scores ranging from 0 to 24, where a higher score signifies increased severity of FOG symptoms.

**Figure 2:**
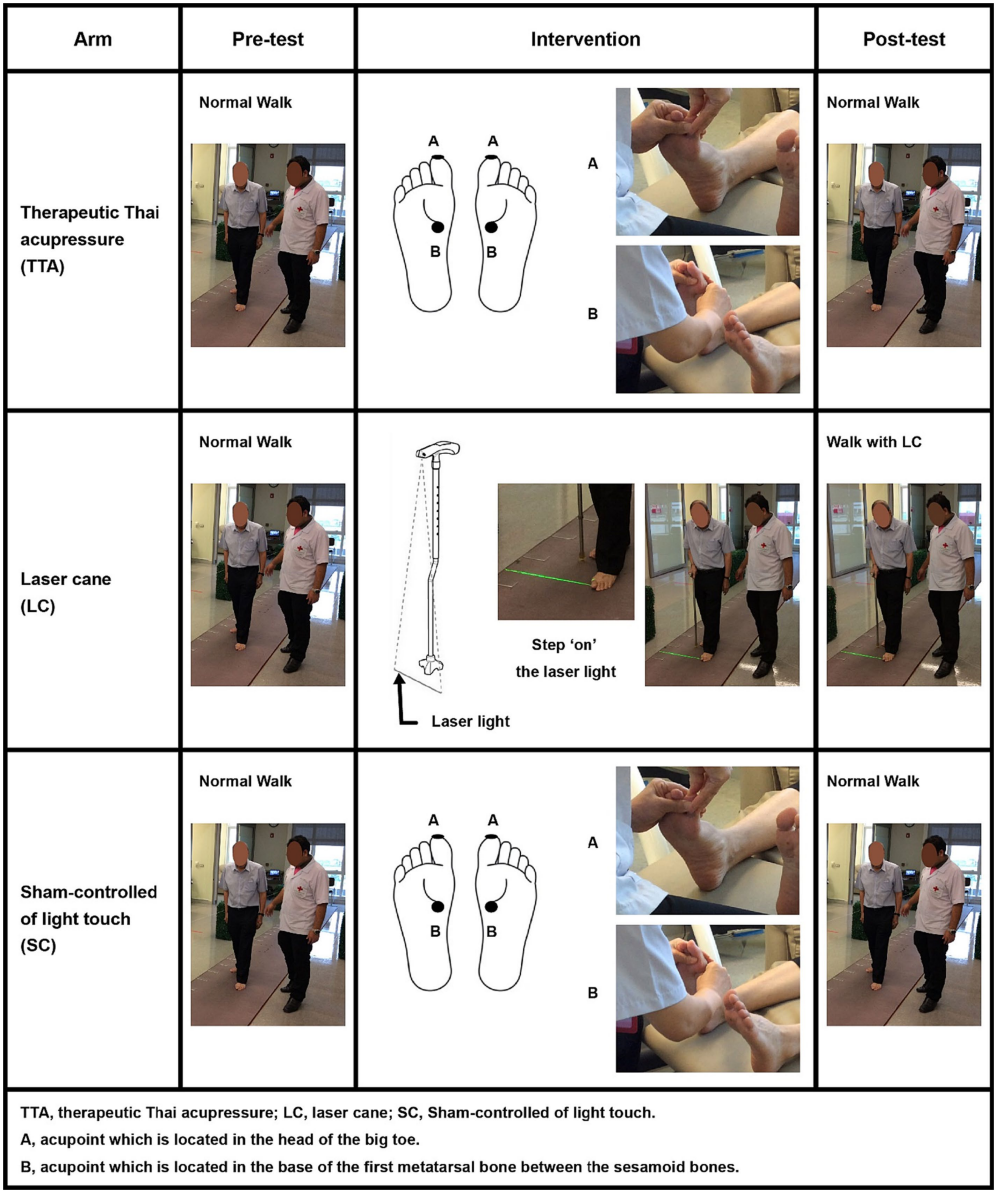
Session flow.

On the randomization day, the participants underwent a single session that comprised a pre-test, the randomized intervention, and an immediate post-test. Each test was facilitated using the GAITRite^®^ software version 3.95 (CIR Systems, Inc., New Jersey, United States), and the participants walked a 10 m hallway at their own pace, starting and stopping 1 m away from the mat to negate recording phases of acceleration and deceleration (36). Mobility was gauged using the timed up and go (TUG) test, which entailed standing from a chair, walking 3 m, turning, walking back, and sitting (37). This test was performed twice with averages used for subsequent analyses. Notably, only the post-test in the LC arm utilized the LC. A 10 min interval was allowed between tests for participant comfort. Finally, subjective discomfort levels during walking were assessed using the visual analog scale (VAS) at both pre and post-test. The VAS scores ranged from 0 to 10, with 10 indicating the most severe discomfort (38) (Figure 2).

### 2.3 Intervention

The intervention procedure encompassed four distinct stages: initial rest and vital signs checks, preparation, the intervention itself, and concluding with rest and vital signs checks. The initial and concluding stages were identical across all study arms, involving a 10 min rest period where participants sat on a chair. Vital signs checks, predominantly focused on blood pressure measurements, were carried out during these stages. Following the completion of the intervention, participants could depart, provided their condition remained stable.

#### 2.3.1 Therapeutic Thai acupressure treatment arm

This study defined TTA as deep acupressure applied to specific acupoints using the thumb (26, 28, 29). In this study, the pressure intensity was gradually increased within 3–5 s until the participants reported mild discomfort, and this pressure level was sustained for 6 s, after which it was gradually reduced over roughly 5 s (26, 28, 29, 39). This procedure was executed by YM, the principal investigator and a certified therapeutic Thai massage practitioner accredited by the Ministry of Education, Thailand, and with 18 years of professional experience.

In the preparation stage, participants’ feet were sanitized using alcohol. For the intervention, acupressure was applied to four predetermined acupoints, split between both feet: the head of the big toe and the base of the first metatarsal bone, located between the sesamoid bones (21, 22, 40). These acupoints were selected based on their significant vibratory and touch pressure sensitivity thresholds in patients with PD, as well as the emergence of the monosynaptic reflex in the tibialis anterior muscle (21, 22, 40). The chosen acupoints aligned with the standard therapeutic Thai acupoints on the plantar, which are recognized for their role in influencing motor function in the lower extremities (25, 26). Adhering to Thai traditional medicine principles, acupressure was initially applied to the left foot before shifting to the right (26). This procedure was repeated four times (21, 22, 40). In total, TTA took 96 s which consisted of 6 s at the discomfort threshold per acupoint, i.e., on 4 target acupoints in total with 2 acupoints per foot, and repeated 4 times (21, 22, 40) (Figure 2).

#### 2.3.2 Laser cane treatment arm

An LC is a specialized walking aid that emits laser light to assist foot placement (14, 31). This study defined LC treatment as using a laser line to which participants attempted to step “on,” not “over” (31). In this study, the LC used was designed by the ChulaPD team. This LC has been widely distributed nationwide with support from the Thai Red Cross Society and the Ministry of Social Development and Human Security. Importantly, this LC is specifically tailored for patients with PD with FOG (14, 16).

During the preparation stage, the height of the LC was first calibrated to each participant, and then the participants were briefed on its usage. For the intervention, participants underwent a 5 min training session walking with the LC. They were given the flexibility to use the cane on either their dominant right or left side. The orientation of the laser was adjusted forward, and the participants were instructed to step comfortably on the trajectory of the laser (Figure 2).

#### 2.3.3 Sham-control of light touch treatment arm

This study defined the SC treatment as involving light touch, without any pressure, applied to the acupoints on the plantar surface using the thumb and sustained for 6 s per acupoint (21, 22, 40). This method was executed by the same practitioner responsible for the TTA treatment. All procedural steps were the same as those in the TTA arm, except the third step wherein light touch was applied instead of TTA. In total, SC took 96 s which consisted of 6 s per each acupoint, i.e., on 4 target acupoints in total with 2 acupoints per foot, and repeated 4 times (21, 22, 40). This method ensured that no reflex withdrawal was triggered, eradicating potential confounding effects (21, 22, 40) (Figure 2).

### 2.4 Outcomes

The primary non-inferiority outcome was stride length. The secondary outcomes included the number of FOG episodes, velocity, double support time, step length, cadence (1, 11), TUG results, and VAS scores. In this study, a step within a FOG episode was defined by each individual’s objective spatial-temporal gait evaluated using GAITRite, which fulfilled two conditions: a double support time exceeding 1.65 standard deviations (SD) above the average and a velocity falling below 90% of the average (41). Improvement in these parameters is denoted by increased stride length, velocity, cadence, and step length and by reductions in FOG episodes, double support time, TUG results, and VAS scores.

### 2.5 Statistical analysis

The sample size was determined using the standard formula specific to non-inferiority trials (42). Based on an assumption that the SD change in stride length from the pre-test to the post-test in the LC arm was 15 cm (14), treatment was deemed non-inferior if the lower limit of the 95% confidence interval (CI) for the mean change in pre- and post-test stride length was above −10 cm in the comparison between the TTA arm and the LC arm. To achieve an 80% statistical power and determine non-inferiority at a one-sided 5% significance level, 28 participants were recruited for each group, subsequently expanded to 30 participants per group. The SC arm served as a reference point to compare against the two active intervention arms.

Baseline characteristics were organized by treatment arm. Continuous data were presented as the mean (SD) and compared by the one-way analysis of variance test, while categorical data were presented as n (%) and compared by the chi-square test. Any duplicate pre- and post-test results were consolidated into an average value. For the primary outcome, the main analysis computed the mean change difference from the baseline (pre-test) to post-test in the TTA arm compared to that in the LC arm, assessing non-inferiority based on the 95% CIs. For the secondary outcomes, the mean difference (95% CI) pre and post-test for each factor in the TTA versus LC arms was calculated using an independent t-test with a two-sided 5% significance level. Additional formal comparisons of the primary and the secondary outcomes aimed to determine the mean difference (95% CI) between each active intervention arm and the SC arm. The analysis method was the intention-to-treat. A *p*-value less than 0.05 was considered statistically significant. All statistical analyses were performed using SPSS (version 22.0; IBM Corp., Armonk, NY, United States) and Stata 16.1 (StataCorp., College Station, TX, United States).

## 3 Results

### 3.1 Participant characteristics

In total, 97 participants were enrolled from March 2020 to August 2021. Among them, 7 participants were either ineligible or declined participation due to time constraints. The remaining 90 patients were randomly allocated to the TTA, LC, and SC arms: 30 participants per arm. All participants complied with the protocol, as shown in Figure 1. All clinicodemographic characteristics were uniformly distributed across the study groups. Overall, 50% (45/90) of the participants were females, and the mean age was 67.5 (8.6) years. The mean UPDRS-III and FOG-Q scores were 19.2 (5.1) points and 9.4 (3.6) points, respectively. There were no significant differences among the 3 arms regarding demographic and disease-related characteristics. The participant characteristics are shown in Table 1. No adverse events occurred.

**Table 1:** Baseline characteristics.

	Total (*n* = 90)	Arm 1: TTA (*n* = 30)	Arm 2: LC (*n* = 30)	Arm 3: SC (*n* = 30)	*p*-value
**Demographic characteristics**					
Gender (male)	45 (50%)	15 (50%)	15 (50%)	15 (50%)	1.00^α^
Age (year)	67.5 (8.6)	68.1 (9.7)	68.5 (8.2)	65.9 (7.7)	0.42^β^
Height (cm)	160.9 (8.5)	161.1 (10.0)	160.6 (7.2)	161.2 (8.3)	0.95^β^
Weight (kg)	57.1 (11.4)	56.0 (9.8)	59.1 (11.7)	56.4 (12.6)	0.52^β^
BMI (kg/m^2^)	21.9 (3.6)	21.5 (3.0)	22.7 (4.3)	21.5 (3.4)	0.42^β^
**Disease-related characteristics**					
PD duration (year)	10.1 (4.9)	9.8 (4.4)	10.4 (5.3)	10.0 (5.1)	0.87^β^
LED (mg)	810.5 (369.0)	826.2 (349.6)	788.7 (412.9)	816.7 (352.7)	0.93^β^
H&Y: ON-state (point)	2.5 (0.5)	2.5 (0.5)	2.5 (0.5)	2.5 (0.5)	0.88^β^
UPDRSIII: ON-state (point)	19.2 (5.1)	19.7 (4.7)	18.7 (4.9)	19.3 (5.7)	0.74^β^
FOG-Q: ON-state (point)	9.4 (3.6)	9.5 (2.9)	9.4 (3.9)	9.3 (4.0)	0.97^β^

Data are presented as mean (SD) or *n* (%) unless otherwise specified. TTA, therapeutic Thai acupressure; LC, laser cane; SC, sham-control of light touch; H&Y, Hoehn & Yahr stage; BMI, body mass index; UPDRSIII, motor section of the Unified Parkinson’s Disease Rating Scale; LED, levodopa equivalent dosage; FOG-Q, freezing of gait questionnaire; ON-state, period during the dopaminergic medication is fully working. ^*^Statistical significance at *p* < 0.05 (two-sided); α: chi-square test; β: one-way analysis of variance test.

### 3.2 Primary non-inferiority outcome

**Figure 3:**
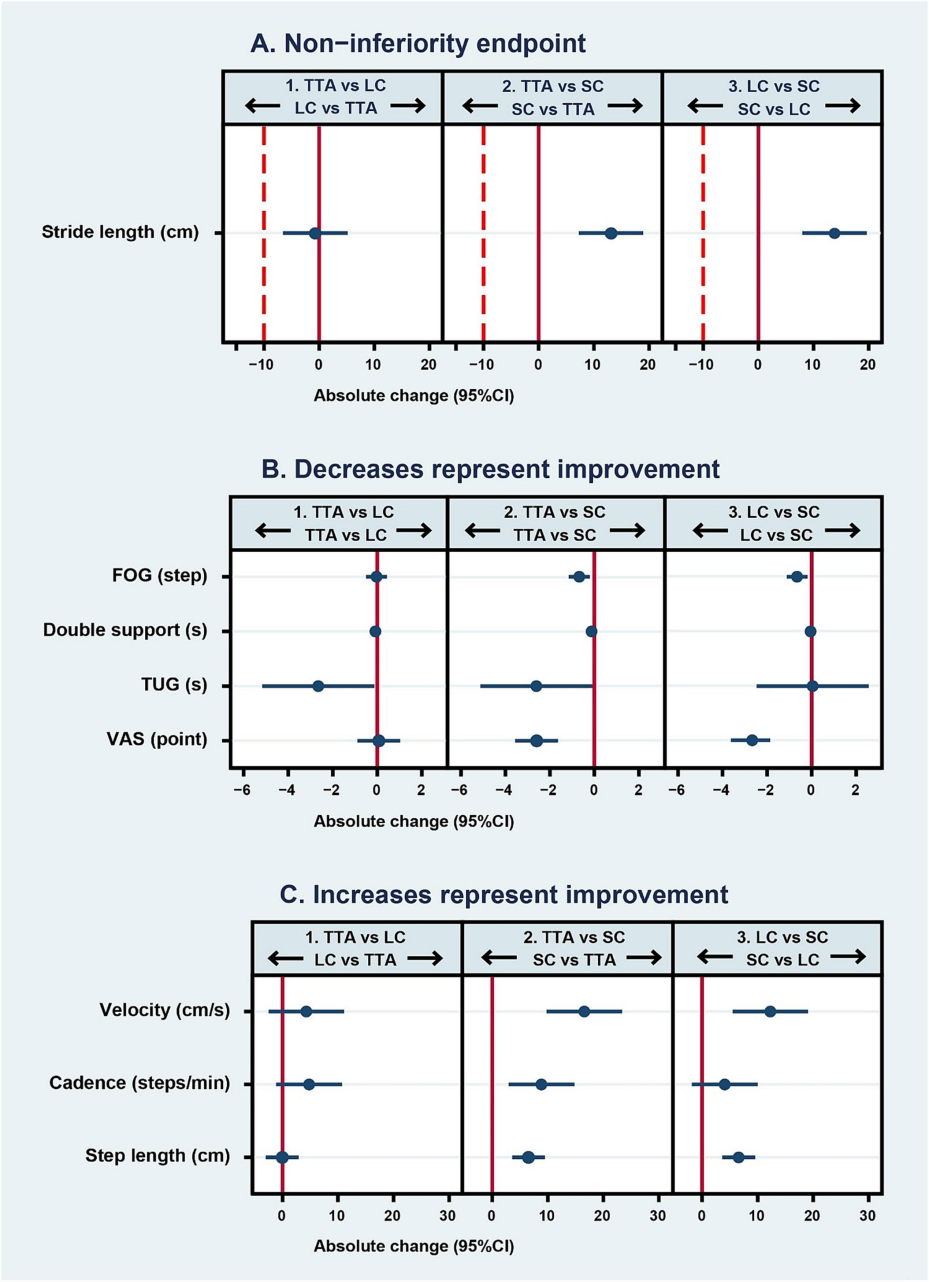
Mean differences (95% CI) in pre- to post-intervention parameter changes between randomized arms. In graph **(A)**, the solid line represents the no-effect level, and the dashed line is the non-inferiority margin. In panel 1, estimates to the left of the dashed line favor the LC arm and those to the right favor the TTA arm. In panels 2 and 3, estimates to the right of the dashed line favor the TTA and LC arms, respectively. In graph **(B)** where decreased scores represent improvement, the solid line represents the no-effect level. In panel 1, estimates to the left of the solid line favor the TTA arm. In panels 2 and 3, estimates to the left of the solid line favor the active intervention arm (TTA and LC, respectively) over the sham comparator arm (SC). In graph **(C)** where increased scores represent improvement, the solid line represents the no-effect level. In panel 1, estimates to the right of the solid line favor the TTA arm over the LC arm. In panels 2 and 3, estimates to the right of the solid line favor the active intervention arm (TTA and LC, respectively) over the sham control (SC). Arrows represent the direction of effect that would be favorable for each arm. TTA, therapeutic Thai acupressure; LC, laser cane; SC, sham-control of light touch.

The average stride length difference pre- and post-test between the TTA and LC arms was −0.7 cm (95% CI, −6.55 to 5.15) (*p* = 0.41, one-sided), satisfying the non-inferiority criteria. The 95% CI indicated a marginally better stride length improvement in the LC arm. In addition, both the TTA and LC arms showed similar improvements when compared to the SC arm, with an increase of 13.11 cm (95% CI, 7.26 to 18.96) (*p* < 0.001) for the TTA arm and 13.8 cm (95% CI, 7.96 to 19.65) (*p* < 0.001) for the LC arm. The details are presented in Table 2 and Figure 3A (33).

**Table 2:** The pre- and post-test mean and SD gait parameter estimates, and pairwise comparisons of the mean difference in change from baseline (95% CI) for TTA versus LC arms, and the TTA and LC arms versus the SC arm.

	Arm 1 (TTA) (*n* = 30)	Arm 2 (LC) (*n* = 30)	Arm 3 (SC) (*n* = 30)	TTA vs. LC		TTA vs. SC		LC vs. SC	
	Mean (SD)	Mean (SD)	Mean (SD)	Mean change difference (95% CI) from pre- and post-intervention	*p*-value	Mean change difference (95% CI) from pre- and post-intervention	*p*-value	Mean change difference (95% CI) from pre- and post-intervention	*p*-value
**Primary non-inferiority outcome**									
*Stride length (cm)*									
Pre	67.00 (17.52)	64.72 (13.66)	67.51 (14.83)						
Post	81.90 (21.60)	80.32 (17.65)	69.31 (18.46)	−0.7 (−6.55 to 5.15)	0.81	13.11 (7.26 to 18.96)	<0.001^*^	13.8 (7.96 to 19.65)	<0.001^*^
**Secondary outcomes**									
*FOG (step)*									
Pre	1.48 (0.76)	1.47 (0.67)	1.40 (0.76)						
Post	0.77 (0.41)	0.78 (0.57)	1.37 (0.87)	−0.03 (−0.5 to 0.44)	0.89	−0.68 (−1.15 to −0.21)	0.005^*^	−0.65 (−1.12 to −0.18)	0.007^*^
*Double support (s)*									
Pre	0.53 (0.21)	0.53 (0.13)	0.52 (0.22)						
Post	0.38 (0.14)	0.47 (0.19)	0.50 (0.27)	−0.09 (−0.15 to −0.02)	0.01^*^	−0.13 (−0.19 to −0.06)	<0.001^*^	−0.04 (−0.11 to 0.03)	0.23
*Velocity (cm/s)*									
Pre	49.30 (15.43)	44.96 (9.80)	48.99 (18.21)						
Post	68.97 (20.75)	60.33 (16.72)	52.07 (20.43)	4.3 (−2.49 to 11.09)	0.21	16.59 (9.8 to 23.38)	<0.001^*^	12.29 (5.5 to 19.08)	0.001^*^
*Cadence (steps/min)*									
Pre	89.33 (15.75)	85.0 (11.61)	86.71 (18.83)						
Post	101.54 (13.22)	92.41 (20.05)	90.06 (18.98)	4.8 (−1.13 to 10.73)	0.11	8.87 (2.94 to 14.8)	0.004^*^	4.07 (−1.85 to 10)	0.18
*Step length (cm)*									
Pre	33.11 (8.69)	32.04 (6.80)	33.43 (7.29)						
Post	40.55 (10.71)	39.52 (9.13)	34.33 (9.10)	−0.05 (−3 to 2.91)	0.97	6.54 (3.59 to 9.49)	<0.001^*^	6.59 (3.63 to 9.54)	<0.001^*^
*TUG (s)*									
Pre	23.30 (7.20)	23.69 (6.21)	23.48 (11.12)						
Post	19.44 (7.59)	22.48 (5.26)	22.22 (9.05)	−2.65 (−5.17 to −0.14)	0.04^*^	−2.61 (−5.12 to −0.09)	0.04^*^	0.05 (−2.47 to 2.57)	0.97
*VAS (point)*									
Pre	6.97 (1.52)	7.03 (1.13)	6.57 (1.33)						
Post	3.63 (1.69)	3.63 (1.79)	5.83 (1.56)	0.07 (−0.89 to 1.03)	0.89	−2.6 (−3.56 to −1.64)	<0.001^*^	−2.67 (−3.63 to −1.71)	<0.001^*^

TTA, therapeutic Thai acupressure; LC, laser cane; SC, sham-controlled light touch; FOG, freezing of gait; TUG, timed-up-and-go test; VAS, visual analogue scale; Pre, pre-test; Post, post-test. ^*^Statistical significance at *p*-value of <0.05 (two-sided), independent *t*-test between 2 arms.

### 3.3 Secondary outcomes

In the study, researchers compared the effect of three distinct interventions: TTA, LC, and SC. The comparative effects among these arms regarding secondary outcomes were manifested differently.

#### 3.3.1 TTA vs. LC arms

There was a marked improvement in two parameters in the TTA arm. The double support time was reduced by −0.09 s (95% CI: −0.15 to −0.02, *p* = 0.01), and the TUG test results decreased by −2.65 s (95% CI: −5.17 to −0.14; *p* = 0.04). Meanwhile, there was no significant difference between the two arms with respect to the FOG that changed by −0.03 steps (95% CI: −0.5 to 0.44; *p* = 0.89), velocity that changed by 4.3 cm/s (95% CI: −2.49 to 11.09; *p* = 0.21), cadence that increased by 4.8 steps/min (95% CI: −1.13 to 10.73; *p* = 0.11), step length that decreased minimally by −0.05 (95% CI: −3 to 2.91; *p* = 0.97), and the VAS score that increased by 0.07 points (95% CI: −0.89 to 1.03; *p* = 0.89). Interestingly, although there was no significant difference, the 95% CIs indicated the TTA arm had greater improvements in velocity and cadence. LC use might lead to lesser improvements in cadence, double support time, and TUG than TTA as the TUG test takes longer to complete when using a cane (Table 2 and Figures 3B,C) (43).

#### 3.3.2 TTA, LC vs. SC arms

The improvements in the TTA arm were either comparable or superior to those in the LC arm across various parameters. Both the TTA and LC arms showed significant improvements over the SC arm in FOG, with a mean difference in pre- and post-test values of −0.68 steps (95% CI: −1.15 to −0.21; *p* = 0.005) for TTA and −0.65 steps (95% CI: −1.12 to −0.18; *p* = 0.007) for LC. Similar findings were obtained for velocity, where it was improved by 16.59 cm/s (95% CI: 9.8 to 23.38; *p* < 0.001) in the TTA arm and by 12.29 cm/s (95% CI: 5.5 to 19.08; *p* = 0.001) in the LC arm. Step length was also better in both intervention arms than in the SC arm, with the TTA arm achieving 6.54 cm (95% CI: 3.59 to 9.49; *p* < 0.001) and the LC arm achieving 6.59 cm (95% CI: 3.63 to 9.54; *p* < 0.001). Further, the VAS scores were higher in both the TTA arm by −2.6 points (95% CI: −3.56 to −1.64; *p* < 0.001) and the LC arm by −2.67 points (95% CI: −3.63 to −1.71; *p* < 0.001).

However, in the comparison between the TTA and SC arms, the TTA arm showed significantly better double support time by −0.13 s (95% CI: −0.19 to −0.06; *p* < 0.001), cadence by 8.87 steps/min (95% CI: 2.94 to 14.8; *p* = 0.004), and TUG by −2.61 s (95% CI: −5.12 to −0.09; *p* = 0.04). These parameters were not significantly different between the LC and SC arms (Table 2 and Figures 3B,C).

### 3.4 Duration of intervention efficacy

Although we did not formally evaluate the efficacy duration of the interventions, a post-intervention telephone follow-up interview provided subjective insights. The participants in the TTA and LC arms reported retained effects for around 3–6 h, whereas those in the SC arm reported persistent effects for approximately 1–3 h. Most participants in all arms reported experiencing good sleep, with the remainder sleeping as usual. No adverse events were reported.

## 4 Discussion

In this study, stride length, the primary non-inferiority endpoint indicating FOG, under TTA treatment, was non-inferior to that under LC treatment. The results showed that TTA improves spatial-temporal gait parameters, mobility and reduces ON-FOG to a similar magnitude or even better relative to LC versus SC. These findings are consistent with previous data and establish the potential of the proposed plantar nerve stimulation therapy and LC therapy for managing gait and FOG (14, 21–23).

It is notable that the difference in mean stride length from pre- to post-intervention between the TTA and LC arms barely reached −1 cm, with the lower limit falling above −7 cm, although some researchers might view the non-inferiority margin of −10 cm as overly generous. Additionally, most gait parameters and comfortable level during walk were comparable between the TTA and LC arms, highlighting that the efficacy of TTA is comparable to that of LC in patients with PD experiencing ON-FOG.

This study found that TTA was effective in mitigating ON-FOG. Although our research did not investigate the mechanisms by which TTA exerted its effects, we hypothesize that TTA could stimulate the degenerated mechanoreceptor response. The benefits can be attributed to the greatest thresholds of the vibratory and touch sensitivity areas brought about by cutaneous sensation. Moreover, tapping into deep sensation, TTA could improve proprioceptive deficits, which can be attributed to the activation of the Golgi tendon organs and spindle cells found in the tibialis anterior muscle. Further, by animating cutaneous and joint receptors, it could be hypothesized that TTA can potentially bridge the proprioceptive feedback from the peripheral sensory afferents to the CPGs in the central nervous system (21, 23, 44, 45). Notably, stimulating peripheral regions at the designated four acupoints is linked to increased resting-state functional connectivity (40). This enhancement is mainly observed between cerebral territories pivotal for visuomotor functionality and proprioception, evident in the sensorimotor cortex which is closely associated with anticipatory postural adjustment (APA) (40). These findings indicate that such upward activation might target the thalamus and potentially influence the cerebellar locomotor region (CLR), which is known to play a pivotal role in ON-FOG and is a cornerstone of the compensatory external pathway (3, 22, 40). The current study found that TTA has a certain effect; it augments proprioceptive functional connectivity, allowing for sustained anticipation of body movement and the positions of the body and limbs, even after the TTA is concluded. This proactive approach enriches motor movement planning (46). With improved proprioception, patients can easily combine visual and proprioceptive feedback, optimizing their visuomotor functionality. This improved proprioception, in turn, refines their ability to gauge spatio-perceptual distances accurately, thus, streamlining their gait movement planning and mobility (46, 47), possibly reducing the rigidity associated with FOG (48).

Visual stimulation techniques have been developed to manage diminished proprioception. A common method is laser light, aiding step verification, attention, and initiating a pronounced optic flow. This flow, driven by central and peripheral vision, enhances space perception and movement (15, 47, 49). Furthermore, there is an interesting interaction of visual stimulation with visuomotor cerebellocortical pathways. When activated, these pathways prevent the impaired functionality of the basal ganglia, enabling an alternate compensatory route (31, 49). TTA appears to affect similar overlapping pathways (40). Particularly, the significant correlation between heightened CLR activation and extended stride length indicates that TTA and LC can be beneficial methods for CLR activation (12). This improvement in stride length supports that external stimulation, whether from TTA or LC, might act as the key to unlocking neural pathways that command central motor functions at the non-inferiority level. This activation is pivotal for stimulating the tibialis anterior muscle and refining motor planning, which collectively enhance APA and stride length and mitigate ON-FOG (50). Therefore, TTA might be beneficial for improving proprioception and space perception, and accordingly alleviating ON-FOG. The improved proprioception might possibly alleviate the visual reliance, magnify multisensory capabilities and decelerate disease progression (15, 17–19). However, these benefits are contingent on individual patient conditions and their environments. Given this intricate interrelation, a comprehensive study into the concurrent effects of both TTA and LC is imperative.

Dopaminergic medications, while beneficial, have implications for both dopaminergic and non-dopaminergic neurotransmitter systems. Furthermore, dopaminergic medications might negatively impact proprioception (51–54). Consequently, these drugs can lead to irregular limb movements and ON-FOG manifestation (4). Such effects suggest that these drugs could contribute to FOG, muscle debilitation, balance issues, and postural instability, even in the early stages of PD (8, 10, 51, 55). Our study clarifies the capability of TTA to modify sensory deficits, especially in proprioception, among patients with ON-FOG. Additionally, TTA was observed to improve muscle strength, especially during the ON-state, in patients with PD (30). This improvement highlights the potential of TTA in counteracting ON-state symptoms. Additionally, data from our phone-based follow-up interviews indicate that subjective effects were markedly longer in both active intervention arms than in the SC arm, highlighting the efficacy of active treatments. Furthermore, most participants described comfort levels during and after treatment in both arms. Interestingly, a significant proportion of participants in all three arms reported sound sleep following the interventions. Lower sleep quality in patients with PD is related to decreased CLR and visuospatial functions and is associated with FOG and a decreased response to levodopa (56–58). Future studies could delve deeper into the sustained efficacy and psychological effects of TTA by objective measurements, other freezing symptoms such as hand movements and speech, and ON-state symptoms.

Our study operated on an open-label format, posing blinding challenges. To counter this limitation, we incorporated a sham control, mirroring the TTA arm but without pressure application, to minimize the impact of this limitation. Our approach to replicating the intervention involved a maneuver that applied light touch, which might stimulate the dorsal column and potentially boost sensory processing. However, the sensory enhancement derived from such light touch—without pressure—is subtle and different from that with TTA (40, 46). Furthermore, another potential study limitation is the demographic characteristics of our study cohort, which was exclusively enrolled from a specialized clinic within a singular tertiary referral hospital in Thailand. Expanding the research to encompass diverse populations would be the logical next step.

Taken together, TTA offers an upfront, noninvasive, and safe CAM approach to manage ON-FOG. A significant contribution of our study is the application of traditional therapeutic methods from Thai medicine, tailored to the unique physical and psychological needs of each patient at every treatment session (26, 29, 39). TTA offers patients with PD an opportunity to hone their physical self-awareness while having a therapeutic experience. In our clinic, ON-FOG management involves a multimodal approach that goes beyond dopaminergic medication and includes various non-pharmacological strategies to counter sensory deficits. This approach for ON-FOG management includes vitamin B12 and B6 treatments (59, 60), because nutritional status may be associated with FOG (61); a specialized Parkinson shoe (23); and, notably, the LC (14, 16). Overall, our data support that TTA is a promising modality that can be added to the multimodal treatment for FOG including homecare application.

## 5 Conclusion

TTA, as a noninvasive therapeutic approach, has comparable efficacy to LC for the treatment of ON-FOG in patients with PD. TTA improved stride length and alleviated ON-FOG by a similar magnitude to LC. This indicates that TTA might also reduce visual reliance during walking and increase life quality. Given its noninvasive nature with simple and safe techniques and potential benefits, TTA is considered a CAM option for PD treatment. However, the results should be interpreted in consideration of the study limitations. Further research is needed for a comprehensive insight into the long-term outcomes, efficacy mechanism, effective utilization strategies, and applicability in diverse patient cohorts.

## Data availability statement

The original contributions presented in the study are included in the article. Further inquiries can be directed to the corresponding author.

## Ethics statement

The studies involving humans were approved by Faculty of Medicine, Chulalongkorn University (IRB No. 211/62). The studies were conducted in accordance with the local legislation and institutional requirements. The participants provided their written informed consent to participate in this study.

## Author contributions

YM: Conceptualization, Data curation, Formal analysis, Methodology, Writing – original draft. OP: Data curation, Formal analysis, Writing – review & editing. SK: Data curation, Formal analysis, Writing – review & editing. CA: Project administration, Resources, Writing – review & editing. HT: Visualization, Writing – review & editing. RB: Conceptualization, Funding acquisition, Methodology, Project administration, Resources, Supervision, Visualization, Writing – review & editing.

## Figures and Tables

**Table:** 

APA	Anticipatory postural adjustment
CAM	Complementary and alternative medicine
ChulaPD	Chulalongkorn Centre of Excellence for Parkinson’s Disease and Related Disorders at King Chulalongkorn Memorial Hospital in Bangkok, Thailand
CI	Confidence interval
CLR	Cerebellar locomotor region
CPGs	Central pattern generators
FOG	Freezing of gait
FOG-Q	Freezing of gait questionnaire
LC	Laser cane
OFF-FOG	Freezing of gait that occurs during the waned effect of medication
ON-FOG	Freezing of gait that occurs during the full effect of medication
ON-state	Period during which the medication has taken full effect
PD	Parkinson’s disease
SC	Sham-control of light touch
SD	Standard deviation
TTA	Therapeutic Thai acupressure
TUG	Timed up and go
UPDRS	Unified Parkinson’s Disease Rating Scale
VAS	Visual analog scale
